# Construction of a novel prognostic model for severe fever with thrombocytopenia syndrome patients based on inflammatory indicators

**DOI:** 10.1186/s12879-026-13457-6

**Published:** 2026-05-02

**Authors:** Xu Xiang, Song Li, Yueqing Dai

**Affiliations:** 1 Department of Laboratory Medicine, Tongji Hospital, Huazhong University of Science and Technology, Wuhan, 430030 China; 2 Department of Gastroenterology, Tongji Hospital, Huazhong University of Science and Technology, 1095 Jiefang Ave, Wuhan, 430030 China

**Keywords:** Severe fever with thrombocytopenia syndrome, Prognosis, Inflammatory indicators, Nomogram

## Abstract

**Objective:**

To identify risk factors associated with poor prognosis in patients with severe fever with thrombocytopenia syndrome (SFTS) and develop a prognostic model based on these factors.

**Methods:**

A retrospective analysis was conducted on 207 patients with SFTS admitted to Tongji Hospital from April 1, 2023, to July 18, 2024. Patients were categorized into survival (*n* = 133) and death (*n* = 74) groups based on their prognosis. Univariate and multivariate logistic regression analyses were performed to identify independent predictors of mortality, incorporating demographic characteristics and inflammatory biomarkers measured within 24 h of hospital admission. A nomogram model was constructed using R software based on the regression coefficients of the identified predictors. The model’s discriminative ability was evaluated using the receiver operating characteristic (ROC) curve, with the area under the curve (AUC) and concordance index (C-index) calculated. Internal validation was performed using the Bootstrap resampling method (1,000 iterations).Furthermore, an external validation cohort comprising 55 patients with SFTS admitted to our hospital between August 2024 and February 2025 was retrospectively collected to evaluate the model’s generalizability and stability.

**Results:**

Age, viral load, procalcitonin (PCT), and interleukin-10 (IL-10) were identified as independent risk factors for poor prognosis. A nomogram model incorporating these four factors demonstrated robust predictive performance, yielding an AUC of 0.905 (95% CI, 0.862–0.949; *P* < 0.001).Internal and external validations confirmed the model’s stability and strong prognostic performance in patients with SFTS. Decision curve analysis (DCA) showed that the nomogram yielded a higher net benefit over a wider threshold probability range than previous models in predicting SFTS mortality.

**Conclusion:**

This study provides a novel prognostic model for SFTS patients, which may aid in early risk stratification. However, its clinical utility and generalizability need to be further validated in larger cohorts.

**Clinical trial:**

Not applicable.

## Introduction

Severe fever with thrombocytopenia syndrome (SFTS) is an acute infectious disease caused by the severe fever with thrombocytopenia syndrome virus (SFTSV), also known as the Dabie bandavirus. Clinical manifestations include fever and thrombocytopenia. These symptoms may be accompanied by nausea, vomiting, malaise, loss of appetite, diarrhea, and muscle pain. Critically ill patients may develop skin petechiae, pulmonary hemorrhage, gastrointestinal hemorrhage, impaired consciousness, or even multi-organ failure [1]. The epidemiological profile of SFTS shows significant regional differences across East Asia. In China, the incidence of SFTS varies by location and season. Endemic areas are concentrated in central and eastern rural regions, where agricultural activities frequently intersect with tick habitats [2]. Notably, Hubei Province has reported a marked increase in incidence, especially in the northeastern areas. This rise is linked to the seasonal reproduction of ticks [3]. Liaoning Province experienced a peak incidence rate of 0.35 per 100,000 in 2019, which was attributed to climatic factors such as precipitation and temperature [4].

The mortality burden of SFTS differs significantly from its incidence rate. In certain Chinese studies, the case-fatality rate of SFTS has been estimated at 12.0% [5]. Conversely, South Korean studies have reported a higher case-fatality rate, reaching up to 16.8% [6]. Among elderly patients and those with compromised immune function, the mortality rate can exceed 30% during the early stages of the disease [7]. If central nervous system involvement or multi-organ failure develops, the mortality rate may rise to 44.7% [8]. The mortality rate of SFTS is closely associated with complications such as multi-organ failure, central nervous system abnormalities, and disseminated intravascular coagulation (DIC) [9]. These variations in mortality rates may be attributed to regional differences in medical resources, patient age distribution, and the presence of underlying comorbidities.

The core mechanism underlying fatal outcomes in SFTS involves virus-induced excessive inflammatory responses, characterized by severe dysregulation of inflammatory mediators. Previous studies indicate that key cytokines, including interleukin-1β (IL-1β), interleukin-6 (IL-6), interleukin-10 (IL-10), and tumor necrosis factor-α (TNF-α), are significantly elevated. Their levels correlate strongly with disease severity and predict adverse clinical outcomes [10]. Notably, in fatal cases, inflammatory peaks occur early in the disease course and persist throughout the illness [11]. Existing prognostic models incorporate parameters such as coagulation function (e.g., prothrombin time [PT], thrombin time [TT]) and metabolic markers (e.g., blood urea nitrogen [BUN], bicarbonate levels) [12–13]. However, these markers primarily reflect late-stage complications and exhibit limited discriminative ability (AUC ≈ 0.81). Importantly, no existing model specifically addresses the core pathophysiological mechanism—dysregulated inflammation. To address this gap, we retrospectively analyzed 207 patients with SFTS and developed a prognostic model based on key inflammatory factors, viral load, and age. This model exhibits high predictive accuracy and provides a practical tool for early risk stratification and clinical intervention.

## Patients and methods

### Criteria for patient inclusion and exclusion

We enrolled 207 SFTS patients hospitalized in the Department of Infection and Intensive Care Unit (ICU) from April 1, 2023, to July 18, 2024. The cohort comprised 89 males and 118 females (mean age: 64.30 ± 9.97 years). Inclusion criteria: ① age ≥ 18 years; ② positive SFTSV nucleic acid test results [1]. Exclusion criteria: ① patients with incomplete laboratory data; ②patients diagnosed in outpatient clinics but not hospitalized;③patients with malignant tumors and hematological diseases. This study has adhered to all pertinent national regulations and institutional policies and has been approved by the local ethics committee, with a waiver for informed consent. The ethical review of the study was conducted by the Ethics Committee of Tongji Hospital, Tongji Medical College, Huazhong University of Science and Technology (approval number: TJ-IRB202411077).

### Data collection and categorization

Basic information and laboratory test data of all patients included in this study were collected at admission, including age, sex, and underlying diseases—such as diabetes mellitus (DM), hypertension (HTN), coronary heart disease (CHD), and cerebral infarction (CI). Unhealthy living habits included smoking and drinking. Clinical manifestations included anorexia, nausea, vomiting, abdominal pain, diarrhea, swollen lymph nodes, alteration of consciousness(AOC), temperature, and days from onset to diagnosis. Laboratory tests included white blood cells (WBC), neutrophils (NEUT), lymphocytes (LYM), the ratio of neutrophils to lymphocytes (NLR), red blood cells (RBC), hemoglobin (HGB), platelet (PLT), interleukin-1β (IL-1β), interleukin-2R (IL-2R), interleukin-6(IL-6), interleukin-8(IL-8), interleukin-10(IL-10), tumor necrosis factor-α (TNF-α), C-reactive protein (CRP), procalcitonin (PCT), ferritin (FERR), and SFTSV load. The 28th day after admission was set as the observation window. Patients who died within this period were categorized in the death group, while those who continued to survive were categorized in the survival group.

### Instruments

Blood routine-related indexes were detected by SYSMES XE2100 hematology analyzer; biochemical indexes such as CRP, PCT, and FERR were detected by Roche C8000 biochemical analyzer; cytokine levels of IL-1β, IL-2R, IL-6, IL-8, IL-10, and TNF-α were detected by Solem auto-chemiluminescence analyzer; and the SFTSV load was detected by the Xi’an Tianlong PANA9600s extractor and GENTIER 96E amplifier. The amplification reagents were produced by Zhongshan Daan Gene Co. The experimental procedures were performed according to the instruction manual.

### SFTSV load quantification

Viral RNA was extracted from 200µL of serum using the PANA9600s automated extraction system with magnetic bead-based kits, followed by quantitative reverse transcription-polymerase chain reaction (qRT-PCR) analysis on the GENTIER 96E amplification platform under optimized cycling conditions: initial reverse transcription at 50 °C for 15 min; pre-denaturation at 95 °C for 15 min; and 45 amplification cycles consisting of denaturation at 94 °C for 15s and annealing/extension at 55 °C for 45 s.The primers and probes targeting the L, M, and S segments of the SFTSV genome were sourced from prior research [14]. For the quantitative PCR assay, the cut-off cycle threshold (Ct) value was established at 35 cycles.

### Statistical methods

As a retrospective observational study, we included all SFTS patients who met the predefined criteria and were admitted during the study period, yielding 74 outcome events for the multivariate model. With four predictors entered into the multivariate model, the Events Per Variable (EPV) ratio was 18.5, well above the recommended minimum of 10. Data were analyzed using SPSS 20.0 statistical software. The Shapiro-Wilk test was used to test the normality of the data. Normally distributed data were expressed as mean ± standard deviation, and skewed data were expressed as median (quartiles) M (P25, P75). The independent samples t-test was used for comparison of normally distributed data, and the Mann-Whitney U-test was used for comparison of skewed data. The χ² test was used to compare the two-component ratios; Receiver operating characteristic (ROC) curves were plotted, and sensitivity, specificity, and area under the curve (AUC) were calculated. Binary univariate and multivariate logistic regression analyses were used, and the goodness of fit of the model was assessed using the Hosmer-Lemeshow test. The independent risk factors obtained were used to establish a nomogram model using the R language (4.3.1). Internal validation was performed using the Bootstrap method, and the predictive power of the model was verified using the C index, calibration curves, and ROC curves. *P* < 0.05 was considered statistically significant. The clinical practicability of the prediction model was assessed through Decision Curve Analysis (DCA), which evaluates the clinical utility of various decision-making strategies by quantifying the net benefit across a range of threshold probabilities.

## Results

### Demographic and clinical characteristics of patients with SFTS

In total, 207 patients (89 men and 118 women) were enrolled in this study, of whom 74 died, and 133 survived. Comparative analysis showed that the age in the survival group was significantly lower than that in the death group. Additionally, the proportion of CHD and the proportion of AOC were significantly lower in the survival group than in the death group. The differences were statistically significant (*P* < 0.05). See Table 1 (Comparison of demographics between the survival group and the death group in patients with SFTS).

**Table 1:** Comparison of demographics between the survival group and the death group in patients with SFTS

Variable (units), RI	Survival group(*n* = 133)	Death group(*n* = 74)	t/z/χ²value	*P* value
age(years)	61.84 ± 9.72	68.74 ± 8.82	t=-5.030	< 0.001
Sex(male), n (%)	53(39.85)	36(48.64)	χ²=1.502	0.220
DM, n (%)	12(9.02)	9(12.16)	χ²=0.514	0.473
HTN, n (%)	37(27.82)	25(33.78)	χ²=0.806	0.369
CHD, n (%)	5(3.75)	10(13.51)	χ²=6.730	0.009
AOC, n (%)	22(16.54)	33(44.59)	χ²=19.178	< 0.001
CI, n (%)	6(4.51)	8(10.81)	χ²=2.992	0.084
Smoking, n (%)	24(18.04)	15(20.27)	χ²=0.154	0.695
Alcoholism, n (%)	18(13.53)	11(14.86)	χ²=0.070	0.791
anorexia, n (%)	6(4.51)	6(8.10)	χ²=1.126	0.289
nausea, n (%)	26(19.54)	23(31.08)	χ²=3.500	0.061
vomiting, n (%)	29(21.80)	25(33.78)	χ²=3.539	0.060
abdominal pain, n (%)	18(13.53)	6(8.10)	χ²=1.366	0.243
diarrhea, n (%)	64(48.12)	43(58.10)	χ²=1.899	0.168
swollen lymph nodes, n (%)	24(18.04)	10(13.51)	χ²=0.711	0.399
Body temperature(℃)	36.9(36.5,37.5)	36.9(36.5,37.9)	Z=-0.346	0.729
Days from onset to admission (day)	7.0(5.0,8.0)	7.0(5.0,9.0)	Z=-0.218	0.825

Abbreviations: DM, diabetes mellitus; HTN, hypertension; CHD, coronary heart disease; AOC, alteration of consciousness; CI, cerebral infarction; RI, reference interval; n, number

Data presented as median (P25, P75) for non-normally distributed variables; mean ± SD for normally distributed variables.Comparisons of categorical variables were performed using the χ² test.*P* < 0.05 considered significant

### Comparison of baseline characteristics between survival and death groups

No statistically significant differences were observed between the two groups in WBC, NEU, LYM, NLR, RBC, and HGB (*P* > 0.05). However, PLT was significantly lower in the death group than in the survival group. In contrast, CRP, PCT, FERR, IL-1β, IL-2R, IL-6, IL-8, IL-10, and TNF-α were significantly elevated in the death group (*P* < 0.05). See Table 2 (Comparison of baseline characteristics between the survival group and the death group in patients with SFTS).

**Table 2:** Comparison of baseline characteristics between the survival group and the death group in patients with SFTS

Variable (units), RI	Survival group(*n* = 133)	Death group(*n* = 74)	t/z/χ²value	*P* value
Viral load (log₁₀ copies/mL)	3.00(2.07,3.72)	4.60(3.91,5.68)	Z=-7.682	< 0.001
WBC(10^9^ /L),3.5–9.5	3.53(2.11,5.70)	3.52(2.09,6.22)	Z=-0.459	0.647
NEUT(10^9^ /L),1.8–6.3	2.13(1.11,3.90)	2.49(1.30,4.62)	Z=-1.168	0.243
LYM(10^9^ /L),1.1–3.2	0.74(0.45,1.20)	0.61(0.33,1.01)	Z = 1.474	0.141
NLR	3.18(1.54,6.07)	3.74(1.75,9.46)	Z=-1.673	0.095
RBC(10^12^ /L),4.30–5.80	4.27(3.96,4.65)	4.13(3.79,4.62)	Z = 0.827	0.409
HGB (g/L),115.0-150.0	127.47 ± 18.33	128.45 ± 20.78	t=-0.351	0.726
PLT(10^9^ /L),125.0-350.0	50.0(35.0,69.0)	36.0(27.0,54.0)	Z = 3.569	< 0.001
PCT(ng/mL),0.02–0.05	0.18(0.08,0.36)	0.60(0.25,1.24)	Z=-6.423	< 0.001
CRP (mg/L),<1	2.9(1.4,7.7)	5.6(2.5,19.3)	Z=-3.058	0.002
FERR(µg/L),15–150	5442.4(2308.5,12653.0)	19652.0(7597.1,44922.0)	Z=-5.526	< 0.001
IL-1β(pg/mL),<5	5.0(5.0,8.0)	7.4(5.0,12.6)	Z=-3.541	< 0.001
IL-2R(U/mL),223–710	1032.0(770.0,1378.0)	1766.0(1226.0,2280.0)	Z=-6.676	< 0.001
IL-6(pg/mL),<7	22.07(9.94,46.08)	85.33(38.16,228.90)	Z=-6.761	< 0.001
IL-8(pg/mL),<62	19.0(13.8,31.1)	47.4(28.9,129.0)	Z=-6.179	< 0.001
IL-10(pg/mL),<9.1	24.5(11.0,66.9)	114.0(51.1,199.0)	Z=-6.481	< 0.001
TNF-α(pg/mL),<8.1	21.5(15.5,29.3)	43.4(27.1,72.9)	Z=-7.102	< 0.001

Abbreviations: RI, reference interval; n, number; WBC, White Blood Cell; NEUT, Neutrophil; LYM, Lymphocyte; NLR, Neutrophil-to-Lymphocyte Ratio; RBC, Red Blood Cell; HGB, Hemoglobin; PLT, Platelet; PCT, Procalcitonin; CRP, C-reactive Protein; FERR, Ferritin; IL-1β, Interleukin-1β; IL-2R, Interleukin-2 Receptor; IL-6, Interleukin-6; IL-8, Interleukin-8; IL-10, Interleukin-10; TNF-α, Tumor Necrosis Factor-α

Data presented as median (P25, P75) for non-normally distributed variables; mean ± SD for HGB; *P* < 0.05 considered significant

### Correlation analysis of independent variables

**Fig. 1:**
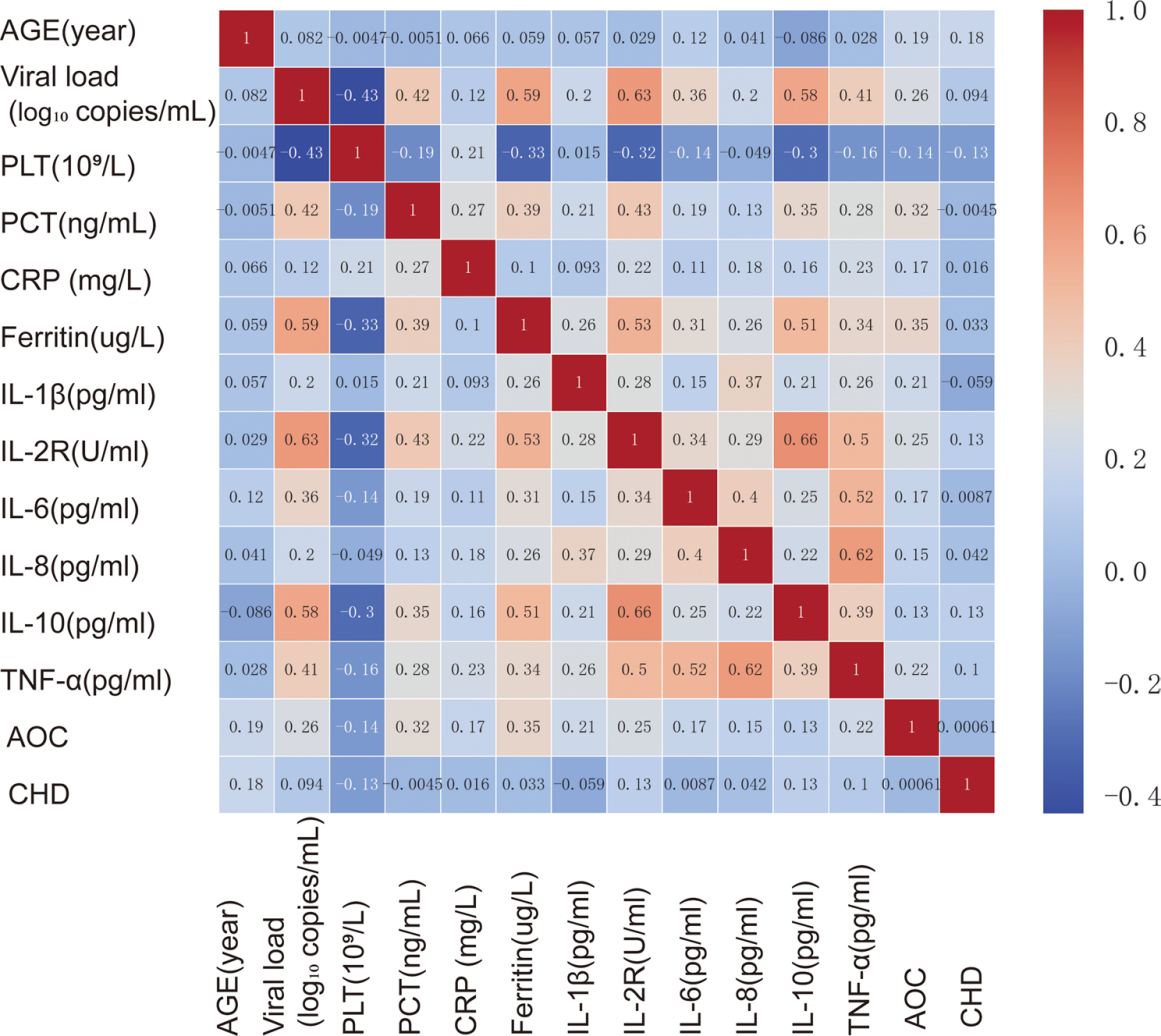
Correlation analysis of the initial screening variables. Note: The intensity of the red color in each cell indicates a stronger positive correlation between the corresponding variables, whereas increasing blue intensity reflects a weaker correlation

Among the 14 independent variables included, Pearson correlation analysis was used to analyze the correlation between the variables, as shown in Fig. 1 (Correlation analysis of the initial screening variables). Notably, the analysis revealed a moderate negative correlation between viral load and PLT (*r*=-0.43) and a moderate positive correlation between PCT and IL-2R (*r* = 0.43). Meanwhile, the variance inflation factor (VIF) testing of all selected variables was < 10, indicating no significant collinearity problem in the model.

### Univariate and multivariate logistic regression analyses of prognostic factors in patients with SFTS

Univariate and multivariate logistic regression analyses were performed with the above 14 variables as independent variables and prognosis as the dependent variable. Backward selection retained four risk factors in the final model: viral load (OR = 2.669, 95% CI 1.764–4.239, *P* < 0.001), age (OR = 1.162, 95% CI 1.102–1.236, *P* < 0.001), PCT (OR = 2.758, 95% CI 1.586–5.231, *P* = 0.001), IL-10 (OR = 1.005, 95% CI 1.001–1.009, *P* = 0.019). See Table 3 (Univariate and multivariate logistic regression analyses of prognostic factors in patients with SFTS). A regression equation was established: Ln(P/1-P) = -15.327 + 0.982 × Viral load + 0.150 × age + 1.015 × PCT + 0.005 × IL-10.

**Table 3:** Univariate and multivariate logistic regression analyses of prognostic factors in patients with SFTS

Variable	Univariable		Full model (14 variables)		Final model (4 variables)	
	OR(95%CI)	*P*-value	OR(95%CI)	*P*-value	OR(95%CI)	*P*-value
Age(year)	1.084(1.047–1.123)	<0.001	1.164(1.099–1.244)	<0.001	1.162(1.102–1.236)	<0.001
Viral load (log₁₀ copies/mL)	3.222(2.311–4.493)	<0.001	2.727(1.622–4.869)	<0.001	2.669(1.764–4.239)	<0.001
PLT(10^9^ /L)	0.976(0.962–0.990)	0.001	1.007(0.987–1.025)	0.471		
PCT(ng/mL)	3.587(2.000-6.433)	<0.001	3.190(1.649–6.767)	0.001	2.758(1.586–5.231)	0.001
CRP (mg/L)	1.022(1.001–1.044)	0.039	0.994(0.961–1.024)	0.680		
FERR(ug/L)	1.000(1.000–1.000)	<0.001	1.000(1.000–1.000)	0.189		
IL-1β(pg/ml)	1.020(1.000-1.041)	0.047	0.978(0.947–1.018)	0.227		
IL-2R(U/ml)	1.002(1.001–1.002)	<0.001	1.000(0.999–1.001)	0.772		
IL-6(pg/ml)	1.004(1.002–1.006)	0.001	1.003(0.999–1.007)	0.203		
IL-8(pg/ml)	1.001(1.000-1.002)	0.034	1.000(0.996–1.002)	0.880		
IL-10(pg/ml)	1.008(1.004–1.011)	<0.001	1.005(1.001–1.010)	0.031	1.005(1.001–1.009)	0.019
TNF-α(pg/ml)	1.022(1.010–1.034)	<0.001	1.001(0.992–1.016)	0.838		
AOC	4.061(2.125–7.760)	<0.001	2.484(0.896–7.010)	0.081		
CHD	4.000(1.312–12.194)	0.015	2.230(0.425–12.50)	0.351		

Abbreviations: OR, odds ratio; CI, confidence interval; PLT, Platelet; PCT, Procalcitonin; CRP, C-reactive Protein; FERR, Ferritin; IL-1β, Interleukin-1β; IL-2R, Interleukin-2 Receptor; IL-6,Interleukin-6; IL-8,Interleukin-8; IL-10,Interleukin-10; TNF-α, Tumor Necrosis Factor-α;CHD, coronary heart disease; AOC, alteration of consciousness

### ROC curve analysis

**Fig. 2:**
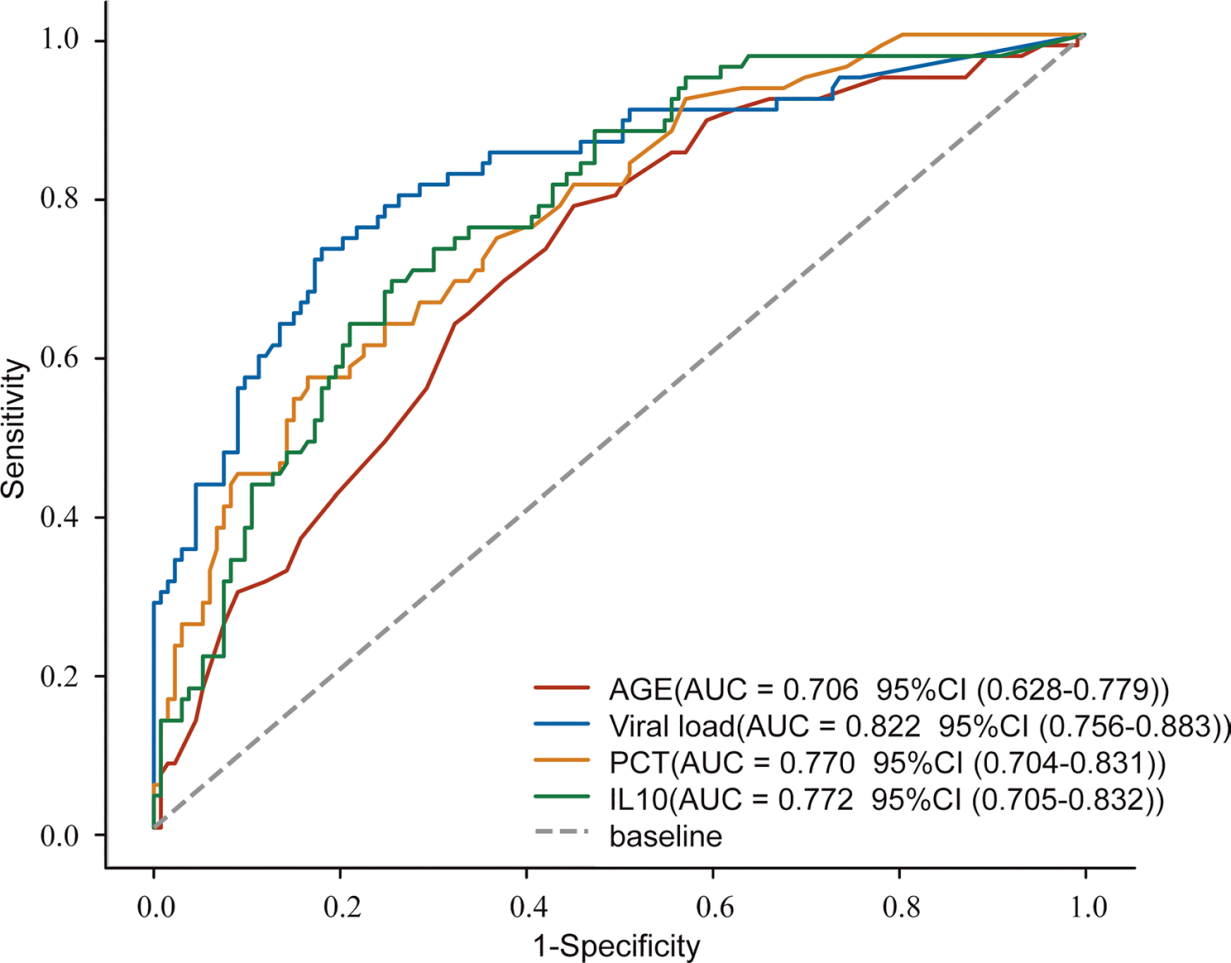
ROC curves comparing individual risk factors significantly associated with prognostic outcomes in patients with SFTS

The ROC curves for each variable of the independent risk factors were plotted individually, as shown in Fig. 2(ROC curves comparing individual risk factors significantly associated with prognostic outcomes in patients with SFTS). Notably, age (AUC = 0.706; 95% CI: 0.628–0.779), viral load (AUC = 0.822; 95% CI: 0.756–0.883), PCT (AUC = 0.770; 95% CI: 0.704–0.831), and IL-10(AUC = 0.772,95% CI: 0.705–0.832) all demonstrated strong diagnostic performance, as presented in Table 4 (Diagnostic performances of age, viral load, PCT, IL-10 and prognostic nomogram model for distinguishing the death group from the survival group in patients with SFTS).

**Table 4:** Diagnostic performances of age, viral load, PCT, IL-10 and prognostic nomogram model for distinguishing the death group from the survival group in patients with SFTS

Variable	AUC (95%CI)	Cut-Off value	Sensitivity (%)	Specificity (%)	Accuracy (%)
Age(year)	0.706 (0.628–0.779)	64.00	78.4%	54.9%	63.3%
Viral load (log₁₀ copies/mL)	0.822 (0.756–0.883)	4.068	73.0%	82.0%	78.7%
PCT(ng/mL)	0.770 (0.704–0.831)	0.510	56.8%	83.5%	73.9%
IL-10(pg/ml)	0.772 (0.705–0.832)	66.60	68.9%	74.4%	72.5%
Prognostic nomogram model	0.905 (0.862–0.949)	0.347	83.8%	85.7%	84.5%

Abbreviations: AUC, area under the curve; CI, confidence interval; PCT, Procalcitonin; IL-10: Interleukin-10

Cut-off values determined by maximizing Youden’s index

### Development of a prognostic risk model for SFTS patients

**Fig. 3:**
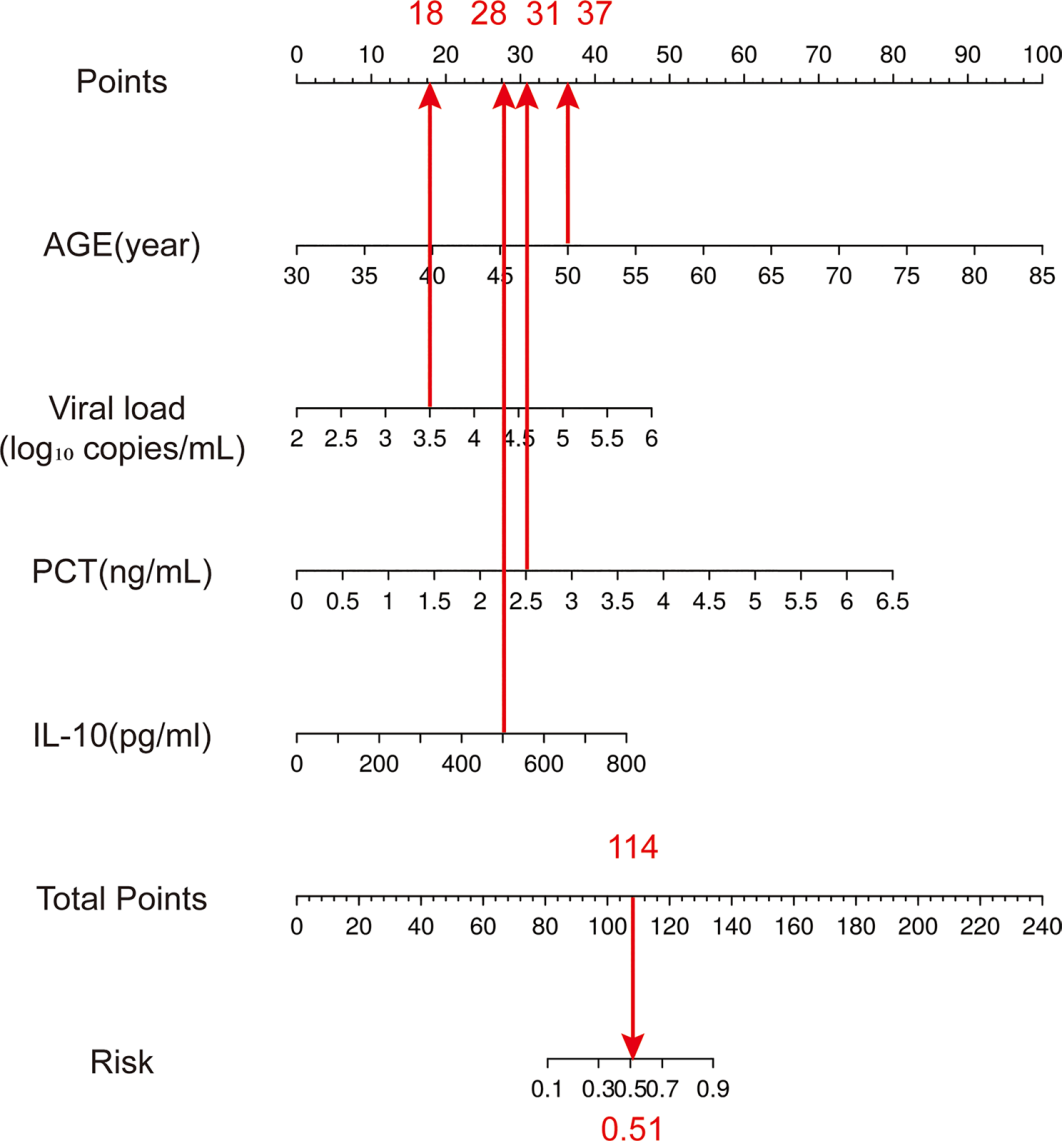
Nomogram model for predicting prognostic risk in patients with SFTS

**Fig. 4:**
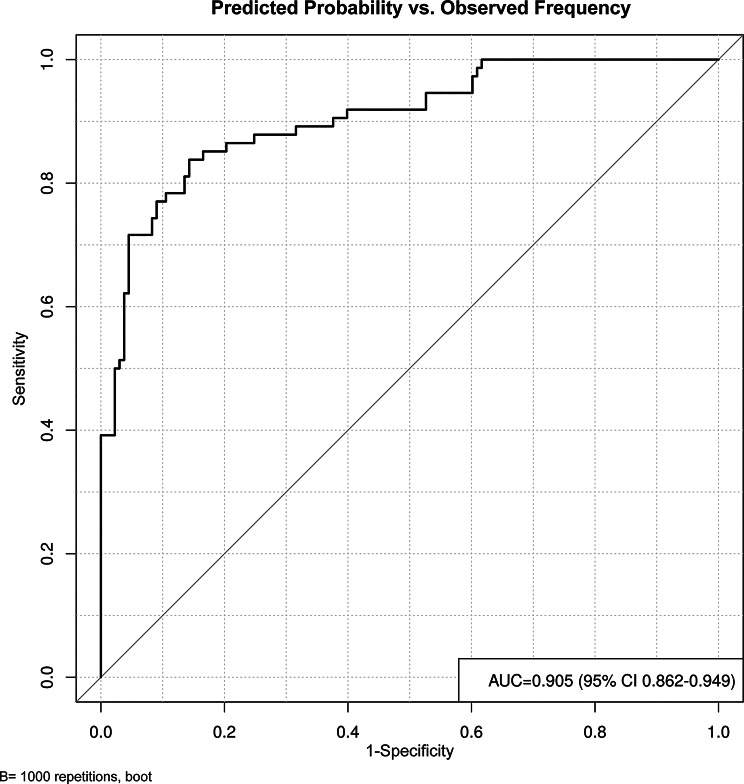
ROC curve evaluating the predictive performance of the nomogram in patients with SFTS

A nomogram model was developed based on independent risk factors identified through logistic regression analysis to predict the prognosis of patients with SFTS, as shown in Fig. 3(Nomogram model for predicting prognostic risk in patients with SFTS). For a hypothetical patient aged 50 years with a viral load of 3.5 log₁₀ copies/mL, PCT of 2.5 ng/mL, and IL-10 of 500 pg/mL, prognostic scores can be calculated using the nomogram by drawing a vertical line from the age axis to the scoring standard axis, yielding a score of 37 points. The scores for viral load, PCT, IL-10, and the total score were 18, 31, 28, and 114, respectively. Drawing a vertical line from the total score axis to the prognosis analysis axis indicated that the probability of poor prognosis was approximately 51%. The threshold value for mortality prediction in the nomogram is 34.7%. This was determined based on the principle of maximizing the Youden index (corresponding to a sensitivity of 83.8%, a specificity of 85.7%, and an accuracy of 84.5%). The patient’s risk of mortality exceeds this threshold, suggesting an elevated likelihood of death. The AUC of the nomogram was 0.905, 95% CI (0.862–0.949), *P* < 0.001. This performance significantly surpasses prior models (AUC range: 0.750–0.813) [10, 12–13]. It is indicated that the constructed nomogram model has good predictive efficacy, as shown in Fig. 4 (ROC curve evaluating the predictive performance of the nomogram in patients with SFTS).

### Internal and external validation

**Fig. 5:**
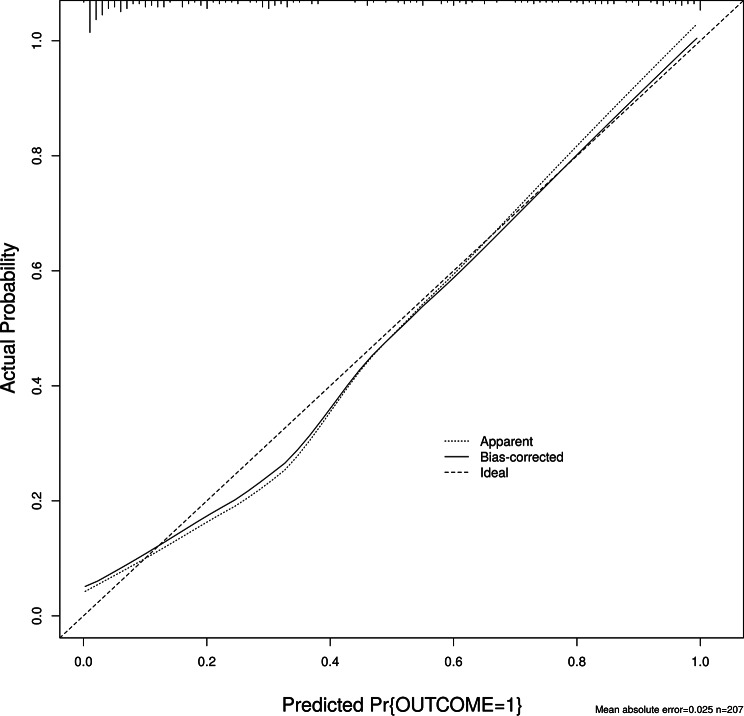
Calibration curve assessing the agreement between predicted and observed prognostic risks in patients with SFTS

In this study, the Bootstrap resampling method was employed for internal validation, with 1,000 iterations conducted to assess the overfitting risk and correct any potential bias in the model. The calibration curve closely approximated the diagonal line, indicating close alignment between predicted probabilities and actual incidence rates. Quantitative assessment revealed a Brier score of 0.12 and a mean absolute error of 0.025, both consistent before and after internal validation, confirming good model calibration and minimal overfitting. The constructed nomogram exhibited a relatively high degree of accuracy, as illustrated in Fig. 5(Calibration curve assessing the agreement between predicted and observed prognostic risks in patients with SFTS). The external validation cohort comprised a total of 55 patients with SFTS, including 36 survivors and 19 nonsurvivors. The mean age was 63.14 ± 10.15 years, and 25 were male (45.5%). The risk probability was calculated using the same variables (age, viral load, IL-10, PCT) as in the modeling stage. The AUC was 0.896 (95% CI 0.836–0.977), showing only a slight decrease compared to the development cohort.

### Comparison with existing models

**Fig. 6:**
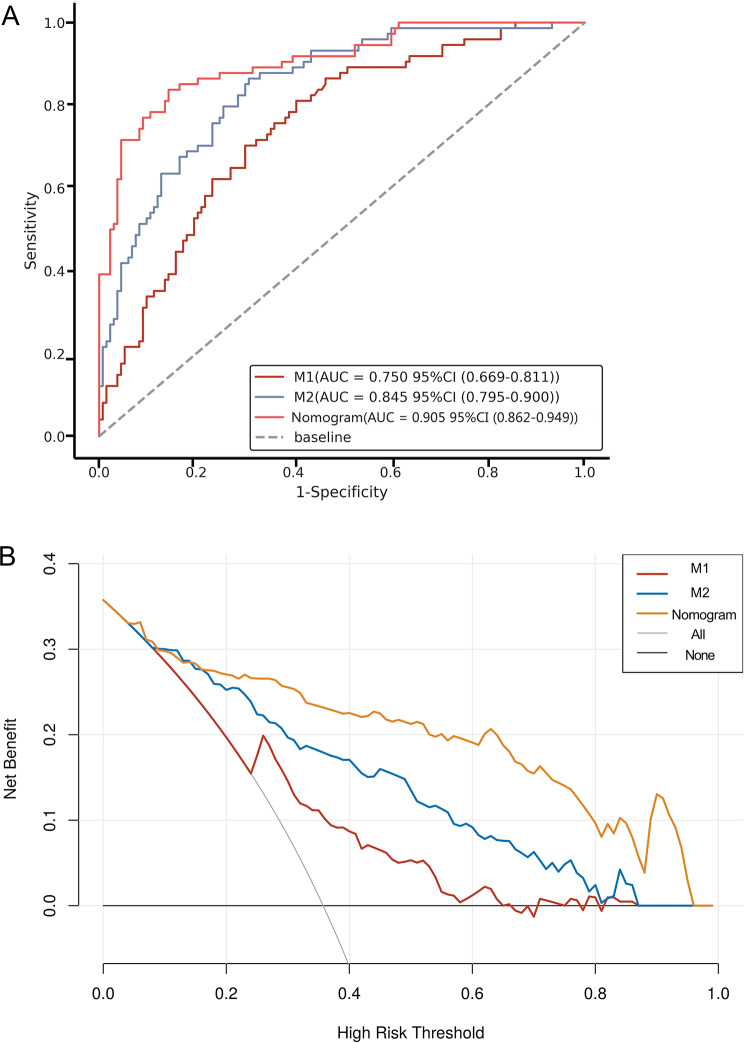
(**A**) Comparison of ROC curves: nomogram versus M1 and M2; (**B**) Decision curve analysis illustrating the net clinical benefit of the nomogram relative to M1 and M2

In our cohort of 207 SFTS patients, we applied the published coefficients from the models developed by Wei X et al. [15] (denoted as M1) and Jia B et al. [16] (denoted as M2).The predicted probabilities were calculated using the following formulas: M1 = 1 / [1 + e^(-(-4.531 + 2.558×AOC + 0.637×(AST/ALT)))] and M2 = 1 / [1 + e^(-(-14.521 + 0.111×Age + 0.245×BUN + 0.089×APTT)))] respectively. The AUC for M1 was 0.750, and for M2 was 0.845, both of which were lower than the AUC of our nomogram (0.905), as shown in Table 5 (Diagnostic performances of M1,M2, and prognostic nomogram model for distinguishing the death group from the survival group in patients with SFTS). Furthermore, the sensitivity (83.8%) and specificity (85.7%) of the nomogram were superior to those of the two aforementioned models (Fig. 6A. Comparison of ROC curves: nomogram versus M1 and M2). DCA was further employed to evaluate the net clinical benefit of the models. The results demonstrated that our nomogram provided a greater net benefit in predicting in-hospital mortality compared to models M1 and M2, and across a wider range of threshold probabilities (Fig. 6B. Decision curve analysis illustrating the net clinical benefit of the nomogram relative to M1 and M2), indicating that the new model has better clinical utility for guiding clinical interventions.

**Table 5:** Diagnostic performances of M1,M2, and prognostic nomogram model for distinguishing the death group from the survival group in patients with SFTS

Model	AUC (95%CI)	Cut-Off value	Sensitivity (%)	Specificity (%)	Accuracy (%)
M1	0.750 (0.669–0.811)	0.058	81.1%	59.4%	69.6%
M2	0.845 (0.795-0.900)	0.294	86.5%	69.2%	78.4%
Prognostic nomogram model	0.905 (0.862–0.949)	0.347	83.8%	85.7%	84.5%

Abbreviations: AUC, area under the curve; CI, confidence interval

Cut-off values determined by maximizing Youden’s index

## Discussion

SFTS is a rapidly progressive acute infectious disease with high heterogeneity in clinical severity [1–2]. While most patients present with mild to moderate symptoms, a subset of individuals deteriorate abruptly and develop fatal complications [7–8]. Early identification of high-risk patients at admission is therefore essential. This allows for timely resource allocation and intensive care, potentially preventing irreversible organ damage.

To further explore the biological mechanisms underlying SFTS progression and prognosis, we performed correlation analysis of key clinical and laboratory indicators. Viral load showed a moderate negative correlation with PLT count (*r* = − 0.43), suggesting that higher viral replication burden is associated with more severe thrombocytopenia. This may be explained by virus-mediated bone marrow suppression and accelerated PLT destruction driven by immune activation and proinflammatory cytokine release [17–18]. In addition, PCT was moderately positively correlated with IL-2R (*r* = 0.43), reflecting coordinated activation of innate and adaptive immune responses in SFTS [19–20]. These findings underscore the complex interactions among viral infection, immune dysregulation, and clinical outcome.

Consistent with previous studies, advanced age and high viral load were identified as independent predictors of fatal outcome in SFTS [21–22]. With advancing age, immunosenescence impairs antiviral immunity and promotes low-grade chronic inflammation. This leads to uncontrolled viral replication and cytokine storm, followed by immune-mediated organ injury and subsequent multiple organ dysfunction [23–24]. Elderly patients are also more vulnerable to secondary bacterial or fungal infections due to virus-induced immune compromise, further elevating mortality risk [25].

Under physiological conditions, PCT is produced by thyroid C cells and remains at low plasma levels (< 0.05 ng/mL). Its elevation is primarily induced by bacterial endotoxins and inflammatory cytokines [26]. However, our data showed relatively low median CRP levels in both groups. This finding implies that PCT upregulation in SFTS patients was mainly driven by virus-induced systemic inflammation rather than superimposed bacterial infection [27]. It also supports the prognostic value of PCT in severe viral infections, even in the absence of overt bacterial co-infection.

Elevated IL-10 has been repeatedly recognized as a key predictor of poor prognosis in SFTS [28–31]. As an anti-inflammatory cytokine, persistently high IL-10 may induce excessive immune suppression, or “immune paralysis,” which impairs viral clearance and indirectly sustains the proinflammatory cascade. IL-10 also contributes to T-cell exhaustion by activating the STAT3 pathway, upregulating inhibitory receptors, and suppressing T-cell proliferation as well as effector function [32–33]. These mechanisms collectively explain the strong association between high IL-10 and fatal outcomes.

The novelty of this study lies in constructing an integrated prognostic nomogram combining age, viral load, PCT, and IL-10. These four factors reflect host susceptibility, viral replication intensity, systemic inflammatory response, and immune regulatory status, respectively. This model directly targets the core virus–inflammation axis of SFTS pathogenesis, which distinguishes it from prior models relying primarily on organ dysfunction or demographic markers [15–16]. Specifically, M1 only contains neurological symptoms and liver enzyme ratios, while M2 only contains age, BUN, and APTT. Neither model targets the key virus-inflammatory axis. Consequently, both exhibit limited predictive efficacy (AUC 0.750 and 0.845, respectively) compared to our nomogram. The resulting nomogram achieved an AUC of 0.905 in the training cohort and 0.896 in external validation. It also demonstrated favorable calibration and net clinical benefit across a wide range of threshold probabilities. Sensitivity and specificity were 83.8% and 85.7%, respectively. By converting a multivariable regression formula into a visual graphical scale, the model improves clinical usability and enables early risk stratification upon admission [34].

In clinical practice, particular attention should be paid to monitoring viral load and inflammatory markers in elderly SFTS patients. We therefore recommend collecting blood samples for complete blood count, inflammatory markers, and viral load within 24 h of admission. The four variables (age, viral load, PCT, and IL-10) are then entered into the nomogram to calculate mortality risk. Patients with predicted mortality risk < 34.7% receive standard supportive care with routine monitoring. In contrast, patients with risk ≥ 34.7% require immediate intensive interventions. These measures include ICU transfer, hemodynamic monitoring, prophylactic antimicrobial therapy, PLT transfusion, and early antiviral therapy. This risk-stratified approach enables early identification of high-risk patients before the critical disease window (days 7–13), optimizing resource allocation and outcomes [22].

This study has several limitations. First, the single-center retrospective design may introduce selection bias. Second, although the sample size was sufficient for model development (EPV = 18.5), it remains relatively modest for broad generalization. Third, only baseline biomarker concentrations were included, and dynamic changes during hospitalization were not analyzed. Of note, emerging evidence has demonstrated that persistently elevated IL-10 is a stronger prognostic predictor than a single time-point measurement [30].

To address these limitations, large-scale multicenter prospective investigations are warranted to validate and optimize the accuracy and stability of the current model. Serial biomarker monitoring (e.g., IL-10 kinetics) will enable construction of a dynamic nomogram for real-time prognostic updating, treatment response assessment, and personalized precision medicine. Further identification of key biological indicators linked to SFTS progression may also improve model performance and clinical applicability.

In conclusion, advanced age, high viral load, elevated PCT, and increased IL-10 were identified as independent risk factors for mortality in patients with SFTS. The inflammatory-marker-based nomogram established in this study demonstrates strong discriminative ability, favorable calibration, and good clinical applicability. This tool can assist clinicians in early risk assessment and support the formulation of personalized treatment plans to improve clinical outcomes.

## Data Availability

The datasets supporting the findings of this study are obtainable from the corresponding author or can be requested via the ethics committee upon reasonable request.

## Abbreviations

SFTS: Severe fever with thrombocytopenia syndrome
SFTSV: Severe fever with thrombocytopenia syndrome virus
ICU: Intensive care unit
DM: Diabetes mellitus
HTN: Hypertension
CHD: Coronary heart disease
CI: Cerebral infarction
AOC: Alteration of consciousness
WBC: White blood cell
NEUT: Neutrophil
LYM: Lymphocyte
NLR: Neutrophil-to-lymphocyte ratio
RBC: Red blood cell
HGB: Hemoglobin
PLT: Platelet
PCT: Procalcitonin
CRP: C-reactive protein
FERR: Ferritin
IL-1β: Interleukin-1β
IL-2R: Interleukin-2 receptor
IL-6: Interleukin-6
IL-8: Interleukin-8
IL-10: Interleukin-10
TNF-α: Tumor necrosis factor-α
qRT-PCR: quantitative reverse transcription-polymerase chain reaction
ROC: Receiver operating characteristic
AUC: Area under the curve
OR: Odds ratio
CI: Confidence interval
DCA: Decision curve analysis
EPV: Events per variable
VIF: Variance inflation factor
DIC: Disseminated intravascular coagulation
PT: Prothrombin time
TT: Thrombin time
BUN: Blood urea nitrogen
AST: Aspartate aminotransferase
ALT: Alanine aminotransferase
APTT: Activated partial thromboplastin time
